# Protein and Metalloprotein Distribution in Different Varieties of Beans (*Phaseolus vulgaris* L.): Effects of Cooking

**DOI:** 10.1155/2017/5957178

**Published:** 2017-02-23

**Authors:** Aline P. Oliveira, Geyssa Ferreira Andrade, Bianca S. O. Mateó, Juliana Naozuka

**Affiliations:** Departamento de Química, Universidade Federal de São Paulo, Diadema, SP, Brazil

## Abstract

Beans (*Phaseolus vulgaris* L.) are among the main sources of protein and minerals. The cooking of the grains is imperative, due to reduction of the effect of some toxic and antinutritional substances, as well as increase of protein digestibility. In this study, the effects of cooking on albumins, globulins, prolamins, and glutelins concentration and determination of Fe associated with proteins for different beans varieties and on phaseolin concentration in common and black beans were evaluated. Different extractant solutions (water, NaCl, ethanol, and NaOH) were used for extracting albumins, globulins, prolamins, and glutelins, respectively. For the phaseolin separation NaOH, HCl, and NaCl were used. The total concentration of proteins was determined by Bradford method; Cu and Fe associated with phaseolin and other proteins were obtained by graphite furnace atomic absorption spectrometry and by flame atomic absorption spectrometry, respectively. Cooking promoted a negative effect on (1) the proteins concentrations (17 (glutelin) to 95 (albumin) %) of common beans and (2) phaseolin concentration (90%) for common and black beans. Fe associated with albumin, prolamin, and glutelin was not altered. In Fe and Cu associated with phaseolin there was an increase of 20 and 37% for the common and black varieties, respectively.

## 1. Introduction

More than 50 varieties of native* Phaseolus vulgaris* L. are present in Latin America, with the common and black bean varieties being the most widely consumed ones in Brazil [1, 2]. In that region, the intake per capita ranges from 1 to 40 kg* per* year [2]. In developed countries, bean consumption is also encouraged because of its health-promoting properties, such as prevention of cardiovascular diseases, obesity, diabetes* mellitus*, and cancer [2, 3].

Beans have great social and economic importance in Brazil being one of the main sources of proteins, plant-derived micronutrients, and minerals for the population [4]. In common beans, the total protein content ranges from 16% to 33%, with high concentrations of aromatic amino acids (lysine, leucine, isoleucine, aspartic acid, and glutamic acid), although low in methionine, cysteine, tryptophan, valine, and threonine [5, 6].

The major protein fractions in* Phaseolus* beans are globulins and albumins, while the minor fractions are prolamins and glutelins [5, 7]. High concentrations of albumins, globulins, and glutelins have been found in raw beans [8]. Salt-soluble globulins, which constitute 34 to 81% of the total protein content, are rich in leucine, lysine, glutamine, and asparagine [5]. The globulin with the highest concentration is phaseolin, whose content corresponds to 40–50% of the total globulins [9, 10].

Phaseolin is a glycoprotein containing neutral sugars, mainly mannose, and consists of three polypeptide subunits with molecular weights between 43 and 53 kDa. Its nutritive value, however, is limited by its low content in sulfur amino acids and its high resistance to enzymatic hydrolysis [3]. Raw phaseolin is highly resistant to in vitro and in vivo digestion, because of glycosylation, which makes its chemical structure rigid and compact. In addition, the low hydrophilic potential of phaseolin limits its accessibility to proteolytic enzymes [2]. Furthermore, the raw albumin and glutelin fractions of the bean proteins show low digestibility; in the case of albumins, this fact is also promoted by a high number of disulfide bridges and the presence of carbohydrates [2].

Low protein digestibility can be improved using thermal treatments, such as domestic cooking [2]. Cooking the bean grains is imperative before intake, since it improves their flavor and palatability and reduces the flatulence factors (raffinose oligosaccharides) and antinutrients (phytic acid and tannins) [3, 4, 11]. However, it is important to consider that cooking can cause considerable changes in the composition of numerous chemical constituents, such as amino acids, vitamins, and minerals. Additionally, studies have shown that cooking may affect the bioavailability of macro- and micronutrients. The digestibility, and hence the absorption of micronutrients such as Fe, is improved by heating processes. With the resultant softening of the food matrix, protein-bound elements are released, thus facilitating their absorption. In addition, heating food alters the inherent factors that inhibit mineral absorption, such as phytates and dietary fiber [8].

In this work, extraction procedures for protein separation and determination of the Fe and Cu concentrations associated with proteins in several* Phaseolus* bean varieties by spectrometric techniques were examined to evaluate the effect of cooking on beans. These studies are essential, because there is a lack of information on the effect of cooking on proteins (albumins, globulins, prolamins, glutelins, and phaseolin) and certain essential elements (Fe and Cu) associated with proteins, especially in the bean cultivars consumed in Brazil (common and black beans).

## 2. Materials and Methods

### 2.1. Reagents and Samples

All solutions were prepared from analytical reagent-grade chemicals and high-purity deionized water obtained from a Milli-Q water purification system (Millipore, Belford, MA, USA).

For the sequential extraction, the following reagents (Merck, USA) were used: acetone, chloroform, ethanol, methanol, NaCl, HCl, and NaOH.

The total protein concentration in the extractants was obtained using Bradford's reagent (BioAgency, Brazil), which was diluted five times with deionized water before analysis. The stock solution used to generate a standard curve was prepared dissolving 4.0 mg of ovalbumin (BioAgency, Brazil) in 2.0 mL of deionized water, using Vortex stirring for 2 min. The solution was then diluted ten times with deionized water.

Analytical-grade Titrisol solutions of 1000 mg L^−1^ of Cu (CuCl_2_) and Fe (FeCl_3_) from Merck were used to prepare the reference analytical solutions to calibrate the instruments.

Two brands of beans comprising seven* Phaseolus* varieties (common, black, rajado, rosinha, bolinha, fradinho, and jalo) were purchased at a local market in São Paulo, each variety weighing 500 g. Six varieties were of the same brand, and the jalo variety was of a different brand. The geographic origin of the varieties was São Paulo (rosinha, rajado, and bolinha) and Minas Gerais (common, black, fradinho, and jalo), according to the producers.

### 2.2. Instrumentation

A ZEEnit 60 model atomic absorption spectrometer (Analytik Jena AG, Germany) equipped with a transversally heated graphite atomizer, a pyrolytically coated graphite tube, and a transversal Zeeman-effect background corrector was used for the determination of Cu and Fe. The spectrometer was operated using a hollow cathode lamp. All measurements were based on integrated absorbance values. The instrumental conditions for the spectrometer and the heating program are shown in Table 1. Argon 99.998%, v/v (Air Liquide Brasil, Brazil), was used as the protective and purge gas.

An atomic absorption spectrometer (Model AAS Vario 6, Analytik Jena AG, Jena, Germany), equipped with a hollow cathode lamp of Fe (259 nm, 4 mA, and 0.8 nm) and a deuterium lamp for background correction, was used. The instrumental parameters (70 L h^−1^ acetylene flow, 430 L h^−1^ air flow, and 8 mm observation height) were stabilized by a fabricant for the determination of Fe in the water, NaCl, ethanol, and NaOH extractants.

Ultrospec 2100 Pro spectrophotometer (Biochrom Ltd, Cambridge, UK), equipped with a xenon lamp and a wavelength range of 190–900 nm, was used for protein determination at 595 nm.

The samples were dried using a freeze dryer (Thermo Fisher Scientific, USA). Samples milling was carried out in a cryogenic grinder (MA 775 model, Marconi, Brazil). The beans were cooked in an electric pressure cooker (Philips Walita Daily Collection, Brazil).

An orbital shaker (Quimis, Brazil) was used to mix the samples and extractants at a rotation velocity of 250 rpm for 30 min. Phase separation was performed by centrifugation (Spectrafuge 6C Compact model, Labnet International, USA).

### 2.3. Preliminary Sample Preparation

One part of raw beans was cleaned with deionized water and dried in an oven at 60°C until a constant mass was obtained. The raw beans were ground in the cryogenic grinder, with 5 min of freezing followed by three cycles of grinding for 2 min, with 1 min of freezing between cycles.

The cooking procedure was adapted from the proposed procedure by Carrasco-Castilla et al. [6]. Raw grains (*ca.* 20 g, not ground) were left to soak in deionized water (*ca.* 200 mL) at room temperature for 24 h. The soaking water was discarded and the soaked beans were cooked in deionized water (beans : water = 1 : 4, w/v) for 30 min. The cooked beans and deionized water were mixed and dried in an oven at 60°C until a constant mass was reached. The dried mixture was ground using a porcelain mortar and pestle, previously decontaminated with 10% v/v HNO_3_.

### 2.4. Sequential Extraction

The sequential extraction procedure has been described by Naozuka and Oliveira [12]. Around 5.0 g of dried raw and cooked grains (all varieties) were used for the solid-liquid sequential extraction with 10 mL of different extractants: a methanol/chloroform mixture (1 : 2 v/v), acetone (75% v/v) [13], deionized water, 0.5 mol L^−1^ NaCl, 70% (v/v) ethanol, and 0.5 mol L^−1^ NaOH: the methanol/chloroform was used for defatting, the acetone was to remove phenols, and the water, NaCl, ethanol, and NaOH were used to produce the four protein fractions examined in the study. The extractions were carried out using an orbital shaker at 1520 ×g for 30 minutes. The separation of the solid phase was carried out by centrifugation at 4000 rpm for 10 minutes. Proteins and Fe were determined, respectively, by the Bradford method [14] and flame atomic absorption spectrometry (F AAS) of the supernatants, except for the methanol/chloroform mixture and acetone fractions.

### 2.5. Phaseolin Separation

Dried and ground raw and cooked grains (common and black varieties, 5.0 g) were subjected to sequential extraction with 10 mL of a methanol/chloroform mixture (1 : 2 v/v) and 10 mL of acetone (75% v/v). The supernatants of these two extractions were discarded. After that, 10 mL of deionized water and 5 mL of NaOH (1.0 M) were added to the resulting solid. To promote protein precipitation, the supernatant was treated with 1 mol L^−1^ HCl until pH 4.5. A mixture of 2 mol L^−1^ NaCl and 1 mol L^−1^ HCl (1 : 20 v/v) was added to the precipitated proteins at pH = 2.0. Four milliliters of deionized water was added to the supernatant at 4°C for phaseolin precipitation. The precipitation was completed after leaving the mixture in a freezer for 24 h. For the extraction procedure, the mixture was gently stirred in an orbital shaker at room temperature (350 rpm for 30 min) and phase separation was achieved by centrifugation (1520 ×g for 15 min).

The total concentration of phaseolin and Cu and Fe associated with phaseolin was obtained with the Bradford method [14] and graphite furnace atomic absorption spectrometry (GF AAS), respectively.

### 2.6. Determination of Total Protein

Protein determination was performed by the Bradford method [14]. Spectrophotometer calibration was performed using analytical reference solutions of 4, 6, 8, 10, 12, 16, and 20 *μ*g of ovalbumin in 1.0 mL of Bradford reagent.

Water, NaCl, ethanol, and NaOH extracts were diluted with deionized water four times (raw and cooked common beans and raw jalo beans) or five times (cooked and raw black, fradinho, rosinha, rajado, and bolinha grains, and cooked jalo grains). The NaOH fractions of rosinha (cooked and raw) and fradinho (raw) were subjected to further dilutions, 10 and 20 times, respectively.

After phaseolin separation, the proteins precipitated with the NaCl/HCl mixture (without phaseolin) and the phaseolin fractions were diluted before analysis. The dilutions were 200 times (raw common and black beans) or 10 times (cooked common and black beans) for the precipitated proteins without phaseolin. The phaseolin fraction was diluted 200 times (raw common beans), 100 times (cooked common and raw black beans), or 10 times (cooked black beans).

### 2.7. Determination of Fe Associated with Albumins, Globulins, Prolamins, and Glutelins in All Phaseolus Bean Varieties by F AAS

Fe determination was carried out by F AAS for the water, NaCl, ethanol, and NaOH extracts. Instrument calibration was performed using analytical solutions with concentrations ranging from 0.5 to 3.0 mg L^−1^ in 0.1% v/v HNO_3_. All the supernatants were previously diluted with deionized water four times (raw and cooked common beans and raw jalo beans) or five times (cooked jalo beans, and raw and cooked black, fradinho, rosinha rajado, bolinha, and common beans). For the glutelin fraction, further dilutions were necessary for the cooked rosinha beans (10 times) and raw fradinho and rosinha beans (20 times).

### 2.8. Determination of Cu and Fe Associated with Phaseolin by GF AAS

Cu and Fe determination was carried out by GF AAS, using the instrument parameters shown in Table 1. A 10 *μ*L aliquot of the analytical solutions or samples (supernatants and precipitated proteins) was introduced into the graphite tube without a chemical modifier and subjected to the heating program described in Table 1. The determination of Cu and Fe in the precipitated proteins (without phaseolin) with NaCl/HCl mixture and in the phaseolin fraction was performed after resuspending the solid in 500 *μ*L of 0.5 mol L^−1^ NaOH.

## 3. Results and Discussion

### 3.1. Determination of Albumins, Globulins, Prolamins, and Glutelins in All Varieties of Phaseolus Beans

Protein quantification was carried out with the Bradford method [14]. The characteristic parameters of the analytical calibration curves (linear range, correlation coefficient (*R*^2^), and sensibility) and the limits of detection (LOD) and quantification (LOQ) are shown in Table 2. The LOD was calculated using the standard deviation of 10 measurements of the analytical blank sample (3 × *σ*_blank_, where *σ* is the standard deviation) and the LOQ was calculated as 3 × LOD. For the sequential extraction, the values were obtained in *μ*g g^−1^, considering a sample mass of 5 g and a final volume of 10 mL.

The separation of lipids and polyphenols was carried out using a mixture of methanol/chloroform or pure acetone [15]. In the absence of lipids and polyphenols, it is possible to separate different protein types by a sequential extraction procedure. The extractants water, NaCl, ethanol, and NaOH were applied sequentially to allow the separation of albumins, globulins, prolamins, and glutelins, respectively [12]. The concentrations of these proteins in the different varieties of* Phaseolus *beans, raw and cooked, are shown in Table 3.

Considering the protein concentrations in Table 3 and applying Student's* t*-test at a 95% confidence limit, it was possible to confirm that cooking changes the distribution of the different proteins. A decrease was found for the majority of the* Phaseolus* bean varieties, particularly for common beans (albumins, globulins, prolamins, and glutelins). Additionally, a negative effect was observed for glutelins in all the varieties. An increase in protein concentration was found for bolinha (albumins, globulins, and prolamins), rajado (albumins, globulins), and jalo (albumins) beans. No effects were observed in the albumins of fradinho beans; the globulins of fradinho and jalo beans; and the prolamins of rosinha and rajado beans.

Globulins and albumins constitute the major proteins fractions in pulse proteins while prolamins and glutelin exist as minor fractions. The raw albumin and glutelin fractions of beans proteins have low digestibility values. For albumins, this fact is related to high number of disulfide bridges. Thermal treatment induces changes in the protein structure that may inactivate antinutritional factors thus increasing the digestibility and biological values of the beans proteins [7]. Previous investigations with common beans showed that the total proteins concentration obtained by the masses sum of all extracts revealed that cooking promoted a sensible decrease in the protein content, mainly in the globulins fraction, when compared to the raw beans [8].

Cooking may therefore promote physical and chemical changes in the proteins, particularly in glutelins, causing variations in the solubility of the proteins. This behavior can be related to changes in the association and dissociation properties of the proteins caused by heating. Protein solubility arises from the thermodynamic equilibrium between protein-protein and protein-solvent interactions and is related to balanced hydrophobic and hydrophilic characteristics of the protein molecules [9, 16–18].

### 3.2. Determination of Fe Associated with Albumins, Globulins, Prolamins, and Glutelins in All Varieties of Phaseolus Beans

The parameters for Fe determination by F AAS have recently been published by our group [19].  Table 4 lists the characteristics of the analytical calibration curves and the LOD and LOQ values.

The Fe concentration in the different proteins is shown in Table 5. It is worth noting that highly diluted supernatant samples were used for F AAS analysis in order to ensure the absence of chemical interferences during Fe quantification.

There was Fe associated with all fractions, both raw and cooked beans. In raw grains, Fe is associated with albumins, prolamins, and glutelins, whereas in cooked grains Fe is linked to albumins and glutelins in most varieties. In addition, variations with respect to the distribution of Fe for the different varieties of beans were observed. The main amino acids in albumins, globulins, prolamins, and glutelins, such as methionine, cysteine, glutamic acid, arginine, aspartic acid, and lysine, are rich in sulfur and charged groups [8, 20–22]. These amino acids have a high affinity toward transition metal ions [23].

Comparing the concentrations in Table 5 and applying Student's* t*-test at a 95% confidence limit, it is possible to conclude that cooking does not cause significant changes in Fe associated with albumins and prolamins for the rosinha, rajado, and fradinho bean varieties. Globulin levels do not exhibit any significant changes after heating for the rajado, rosinha, black, and fradinho beans. Cooking results in a variation of the Fe concentration when associated with glutelins for the black, bolinha, rajado, and jalo beans, since it is possible to observe an increase (180%) and a decrease (78%) for the jalo and rajado beans, respectively.

For common beans, studies showed that, in raw grains, Fe was associated with albumins, globulins, and glutelins and, after the cooking process, Fe was in the albumins and globulins fractions. The main amino acids constituents of albumins, globulins, prolamins, and glutelins are rich in sulfur and charged groups that present high affinity by metal ions [8]. Heating may result in the denaturation of the proteins. In this scenario, interactions between proteins and essential elements can be established or lost [24]. Softening of the food matrix allows certain elements associated with proteins to be released, facilitating their absorption by the human body [19]. Furthermore, during cooking phytate loses phosphate linkages transforming from inositol hexaphosphate to penta-, tetra- or triphosphate and decreases the inhibitory capacity [25]. Therefore, high temperatures modify the composition of antinutrients (e.g., phytate and dietetic fibers) that inhibit the absorption of essential elements [9].

### 3.3. Effect of Cooking on Phaseolin Concentration in Common and Black Beans

The effect of cooking on the phaseolin concentration was studied in the two* Phaseolus* bean varieties most consumed in Brazil: the common and black varieties. With the proposed method, we obtained the characteristic parameters of the analytical calibration curve (linear range (2–20 *μ*g mL^−1^), correlation coefficient (*R*^2^ = 0.9917), and sensibility (0.0106)), as well as the LOD (0.19 *μ*g g^−1^) and LOQ (0.58 *μ*g g^−1^) values.

The concentration values of the precipitated proteins without phaseolin and that of the phaseolin fraction are shown in Table 6. Cooking affects negatively the precipitated proteins (without phaseolin) and the phaseolin fraction for both bean varieties. Regarding the precipitated proteins without phaseolin, the greatest effect is observed for common beans, compared to black beans. The reduction of phaseolin concentration, approximately 90%, is significant for both common and black beans.

Studies have shown that heating promotes a decrease in the total protein concentration, particularly for globulins such as phaseolin [8]. Heating can alter the native conformation of proteins due to disturbances in the noncovalent interactions that stabilize the protein structure, modifying the association and dissociation interactions that occur between amino acids containing opposite charges and/or between protein subunits, altering their isoelectric point and, consequently, their solubility [5, 8, 16]. In the common beans, the secondary structure of the phaseolin is preserved, while its tertiary and quaternary structure suffer alteration, increasing the hydrophilic surfaces that indicate a breakdown of the phaseolin subunit interactions and lead to a higher degree of hydrolysis [2].

### 3.4. Effect of Cooking on the Concentrations of Cu and Fe Associated with Phaseolin in Common and Black Beans

The determination of Cu and Fe associated with phaseolin was carried out by GF AAS. A detector with high sensibility, such as that in GF AAS, is necessary in order to determine Cu and Fe in extracts after the fractionation steps used to isolate phaseolin. The parameters for the determination of Cu and Fe by GF AAS are listed in Table 7.

The concentration of Cu and Fe associated with precipitated proteins (without phaseolin) and that of the phaseolin fraction are shown in Table 8. For common beans, cooking promotes an increase of 91% in the concentration of Fe associated with precipitated proteins (without phaseolin) and a decrease of 24% for Fe associated with phaseolin. The reverse effect is observed for Cu, an increase of 20% in phaseolin after cooking.

For black beans, similar effects were observed to those with common beans. Cooking promoted an increase of 146% in the concentration of Fe associated with precipitated proteins (without phaseolin) and a reduction of 21% of Fe associated with phaseolin. The heating process reduced the concentration of Cu associated with precipitated proteins by 56% and increased it by 37% in the phaseolin fraction.

The presence of Cu and Fe associated with phaseolin may be a result of the existence of aromatic moieties and acid and basic amino acids, such as arginine, histidine, lysine, aspartate, glutamate, and tyrosine, which are responsible for complexation reactions with essential elements. In previous studies with the Jamapa variety, the chelating activity of phaseolin and other low molecular weight proteins toward Cu was confirmed for proteins of low molecular weight. Additionally, the authors showed that the carboxylic groups have a high affinity toward Cu through electrostatic interactions. Both elements of interest have a similar mechanism of interaction with phaseolin; however, the association of Fe with this globulin is weaker than with Cu since heating decreases the Fe concentration in the phaseolin fraction. It is possible to infer that this effect results from a higher coordination number for Fe chelation than for Cu [6].

Moreover, variations in the Cu and Fe concentration can be promoted through interactions of these elements with the matrix components. Fe bioavailability is modified by the presence of tannins, antinutrients that form insoluble complexes when associated with Fe. The tannins concentration varies with the color of the bean shells, being higher in darker seeds. Therefore, the formation of insoluble complexes with Fe is expected to be greater in black beans, resulting in less Fe available for complexation with the proteins. For the two bean varieties, the increase in Fe concentration in the precipitated proteins (without phaseolin) after cooking arises as the heating process decreases the action of the antinutrient compounds that form insoluble complexes with Fe [8, 17, 25]. For Cu, the reduction in the Cu concentration in the precipitated proteins (without phaseolin) can be explained by the formation of Maillard compounds [12, 26, 27]. The processing of foods rich in protein and carbohydrates promotes the development of Maillard reaction, where the Maillard reaction products behave as anionic polymers, forming stable complexes with metal cations such as copper [28].

## 4. Conclusion

Cooking* Phaseolus* bean varieties changes their albumin, globulin (including phaseolin), prolamin, and glutelin distribution, the solubility decreases for all four fractions, and the extent to which solubility was affected varied with the type of protein. Additionally, Fe and Cu metalloproteins undergo changes with heating. Chemical reactions can justify these variations. Studies on the effects of cooking are essential, since cooking beans prior to consumption is necessary; the results of such studies would provide nutritional information that will add value to this food, which is widely consumed by the world's population. Finally, it is important to point out that chemical speciation studies are imperative in food sciences to demonstrate the effect of cooking on the distribution of chemical species that are essential for humans.

## Figures and Tables

**Table 1:** Instrumental parameters and heating program for Fe and Cu determination by GF AAS.

Instrumental parameters	Fe^a^	Cu^b^	
*ƛ*/nm	248.3	324.8	
Slit/nm	0.8	0.8	
I/mA	4.0	4.0	

Heating program			

Step	T/°C	Ramp/°C s^−1^	Hold/s

Drying	130	5	20
Pyrolysis	1200^a^/1000^b^	100	15
Atomization	2300	2300	5
Cleaning	2500	1200	2

**Table 2:** Characteristic parameters of the analytical calibration curve and LOD obtained for proteins determination by Bradford method.

	Linear range (*µ*g mL^−1^)	*R*	*R* ^2^	Sensibility	Analytical blank	LOD (*µ*g g^−1^)	LOQ (*µ*g g^−1^)
Sequential extraction	2–12	0.9935	0.9871	0.049	Water	0.53	1.60
					NaCl	0.55	1.66
					Ethanol	0.56	1.67
					NaOH	0.54	1.62

**Table 3:** Proteins concentrations in different varieties of *Phaseolus *beans (raw and cooked).

Variety	Condition	Concentration (mg g^−1^) ± standard deviation (*n* = 3)			
		Albumins	Globulins	Prolamins	Glutelins
Common	Raw	1.2 ± 0.1	1.2 ± 0.1	1.3 ± 0.1	14 ± 1
	Cooked	0.99 ± 0.01	0.94 ± 0.01	0.98 ± 0.01	0.74 ± 0.01
Black	Raw	1.3 ± 0.1	1.2 ± 0.1	1.2 ± 0.1	2.8 ± 0.2
	Cooked	0.71 ± 0.01	0.76 ± 0.01	0.64 ± 0.01	2.2 ± 0.2
Rosinha	Raw	0.99 ± 0.01	1.3 ± 0.2	0.87 ± 0.01	6.1 ± 0.1
	Cooked	0.92 ± 0.01	0.81 ± 0.01	0.85 ± 0.01	3.1 ± 0.3
Bolinha	Raw	0.77 ± 0.01	0.73 ± 0.01	0.83 ± 0.01	14 ± 1
	Cooked	0.99 ± 0.01	0.94 ± 0.01	0.97 ± 0.01	2.3 ± 0.1
Rajado	Raw	0.95 ± 0.01	0.83 ± 0.01	0.87 ± 0.01	1.9 ± 0.1
	Cooked	1.1 ± 0.1	0.98 ± 0.01	0.85 ± 0.01	0.72 ± 0.01
Fradinho	Raw	1.0 ± 0.1	1.1 ± 0.1	1.04 ± 0.01	6.3 ± 0.1
	Cooked	1.1 ± 0.1	0.98 ± 0.01	0.95 ± 0.01	4.5 ± 0.1
Jalo	Raw	0.72 ± 0.01	1.1 ± 0.1	0.96 ± 0.01	2.4 ± 0.2
	Cooked	1.1 ± 0.1	0.97 ± 0.01	0.89 ± 0.01	1.7 ± 0.1

**Table 4:** Characteristic parameters of the analytical calibration curve, LOD, and LOQ obtained for Fe determination by F AAS.

*λ* (mm)	Linear range (mg L^−1^)	*R* ^2^	LOD (*µ*g g^−1^)	LOQ (*µ*g g^−1^)
259	0.25–3.0	0.9961	0.18 (albumins)	0.54 (albumins)
			0.54 (globulins)	1.62 (globulins)
			1.64 (prolamins)	4.92 (prolamins)
			1.68 (glutelins)	5.04 (glutelins)

**Table 5:** Iron concentration in albumins, globulins, prolamins, and glutelins of* Phaseolus* beans.

Variety	Condition	Concentration (*μ*g g^−1^) ± standard deviation (*n* = 3)			
		Albumins	Globulins	Prolamins	Glutelins
Common	Raw	20 ± 1	4 ± 2	3 ± 1	9 ± 1
	Cooked	25 ± 2	12 ± 3	13 ± 2	8 ± 1
Black	Raw	21 ± 1	10 ± 1	10 ± 1	20 ± 6
	Cooked	30 ± 2	10 ± 2	17 ± 1	50 ± 5
Rosinha	Raw	27 ± 2	10 ± 2	17 ± 1	40 ± 5
	Cooked	30 ± 1	16 ± 4	14 ± 2	40 ± 4
Bolinha	Raw	30 ± 1	11 ± 1	13 ± 1	22 ± 6
	Cooked	25 ± 1	8 ± 1	24 ± 1	36 ± 4
Rajado	Raw	23 ± 1	8 ± 3	11 ± 3	27 ± 4
	Cooked	24 ± 1	6 ± 2	9 ± 1	6 ± 2
Fradinho	Raw	21 ± 1	7 ± 2	11 ± 3	24 ± 13
	Cooked	23 ± 2	7 ± 1	10 ± 3	11 ± 4
Jalo	Raw	22 ± 1	4 ± 1	9 ± 2	12 ± 5
	Cooked	26 ± 1	9 ± 2	15 ± 2	34 ± 9

**Table 6:** Concentrations of precipitated proteins without phaseolin and phaseolin in the common and black beans.

	Concentration (mg g^−1^) ± standard deviation (*n* = 3)			
	Common		Black	
	Raw	Cooked	Raw	Cooked
Precipitated proteins without phaseolin	5.8 ± 0.1	2.64 ± 0.01	3.31 ± 0.04	2.7 ± 0.1
Phaseolin	0.12 ± 0.01	0.013 ± 0.001	0.10 ± 0.01	0.009 ± 0.001

**Table 7:** Characteristic parameters of the analytical calibration curve, LOD, and LOQ obtained for Cu and Fe determination by GF AAS.

	Linear range (mg L^−1^)	*R* ^2^	Sensibility	LOD (ng g^−1^)	LOQ (ng g^−1^)
Cu	10–80	0.9881	0.0043	0.47	1.41
Fe	10–80	0.9956	0.0055	0.05	0.16

**Table 8:** Cu and Fe concentration associated with precipitated proteins without phaseolin and phaseolin.

	Concentration (*μ*g g^−1^) ± standard deviation (*n* = 3)			
	Fe		Cu	
	Raw	Cooked	Raw	Cooked
*Common beans*				
Precipitated proteins without phaseolin	8.9 ± 0.1	17 ± 1	4.2 ± 0.9	2.6 ± 0.4
Phaseolin	3.2 ± 0.1	2.4 ± 0.1	0.72 ± 0.04	0.86 ± 0.03

*Black beans*				
Precipitated proteins without phaseolin	5.6 ± 0.3	14 ± 1	4.1 ± 0.5	1.8 ± 0.1
Phaseolin	2.9 ± 0.1	2.3 ± 0.1	0.84 ± 0.03	1.2 ± 0.1
